# Vascular and Blood Compatibility of Engineered Cationic Cellulose Nanocrystals in Cell-Based Assays

**DOI:** 10.3390/nano11082072

**Published:** 2021-08-15

**Authors:** Alexandre Bernier, Tanner Tobias, Hoang Nguyen, Shreshth Kumar, Beza Tuga, Yusha Imtiaz, Christopher W. Smith, Rajesh Sunasee, Karina Ckless

**Affiliations:** Department of Chemistry, State University of New York at Plattsburgh, Plattsburgh, NY 12901, USA; abern012@plattsburgh.edu (A.B.); ttobi004@plattsburgh.edu (T.T.); hnguy021@plattsburgh.edu (H.N.); skuma010@plattsburgh.edu (S.K.); tuga0002@umn.edu (B.T.); yusha.imtiaz@emory.edu (Y.I.); C.W.Smith022@gmail.com (C.W.S.)

**Keywords:** cellulose nanocrystals, cationic, immunomodulator, hemolysis, cytotoxicity

## Abstract

An emerging interest regarding nanoparticles (NPs) concerns their potential immunomodulatory and pro-inflammatory activities, as well as their impact in the circulatory system. These biological activities of NPs can be related to the intensity and type of the responses, which can raise concerns about adverse side effects and limit the biomedical applicability of these nanomaterials. Therefore, the purpose of this study was to investigate the impact of a library of cationic cellulose nanocrystals (CNCs) in the human blood and endothelial cells using cell-based assays. First, we evaluated whether the cationic CNCs would cause hemolysis and aggregation or alteration on the morphology of red blood cells (RBC). We observed that although these nanomaterials did not alter RBC morphology or cause aggregation, at 24 h exposure, a mild hemolysis was detected mainly with unmodified CNCs. Then, we analyzed the effect of various concentrations of CNCs on the cell viability of human umbilical vein endothelial cells (HUVECs) in a time-dependent manner. None of the cationic CNCs caused a dose-response decrease in the cell viability of HUVEC at 24 h or 48 h of exposure. The findings of this study, together with the immunomodulatory properties of these cationic CNCs previously published, support the development of engineered cationic CNCs for biomedical applications, in particular as vaccine nanoadjuvants.

## 1. Introduction

The medical nanotechnology field has grown exponentially in the past 10 years and the possibilities of applications of cellulose nanocrystals (CNCs) in this context have been expanded from the initial proposed use as drug delivery platforms [1] to sophisticated bio-imaging [2] and pH sensing [3] systems, among others [4]. The source of CNCs is cellulose. This polymer, made of glucose, plays a crucial role in maintaining the structure of the plant cell wall and it is the most abundant polysaccharide on earth [5,6,7,8,9]. CNCs are unique nanomaterial obtained from the acid hydrolysis of native cellulose, forming rigid “rod-like” crystalline nanocellulose (length typically between 100–200 nm and diameter ~5–10 nm). They exhibit remarkable strength and physicochemical properties including a high aspect ratio, low density, and large specific surface area, as well as have the presence of abundant hydroxyl groups for surface chemical modifications [7,10,11]. In fact, the presence of abundant hydroxyl groups allows these nanomaterials to be engineered by modifying these groups with various functional polymers and pendants with the purpose to be tailored for specific biomedical applications [9], including potential immunomodulators. Polysaccharides, such as 2,3-O-acetylated-1,4-β-D-glucomannan, have been shown to elicit an immune response by stimulating the secretion of cytokines, interleukin 1-beta (IL-1β), and tumor necrosis factor alpha (TNF-α) in human cell lines [12]. The immune response is a physiological event that occurs in many biological systems and it is the foundation of vaccine effectiveness. The proper immune response and lasting immunity towards a pathogen elicited by a vaccine is sometimes achieved only in the presence of adjuvants, which are substances that help to boost this response [13,14,15]. Adjuvants currently approved as components of vaccines are particulate nanomaterials such as “alum” (aluminum oxohydroxide and aluminum hydroxyphosphate) or oil-in-water emulsions, which act as both vaccine delivery vehicles and immunostimulants [16,17]. The emerging interest in CNCs for biomedical applications as well as their particulate morphology prompted us to develop a series of wood-based CNCs with positive surface charges and potential immunomodulatory activities that hopefully could be further developed in newly engineered vaccine nanoadjuvants. In fact, the immunostimulatory activity of cationic CNCs was first described in our previous work in which we found that they induced the secretion of the inflammatory cytokine, IL-1β, in mouse and human macrophage cells [18,19]. Recently, we successfully expanded the library of cationic CNCs by engineering the surface of CNCs with poly[2-(methacryloyloxy)ethyl]trimethylammonium chloride (METAC) and poly(aminoethyl methacrylate hydrochloride (AEM)) possessing pendant cationic groups (+NMe_3_ and +NH_3_ respectively)[20]. By changing the proportion of initiators (2-bromoisobutyryl bromide, Brib) and monomers (METAC and AEM) during the polymerization process, we obtained a series of cationic CNCs with different surface charges and hydrodynamic sizes. This new library of cationic CNCs was evaluated using three different cell-based assays and relevant immune cells, including mouse cell lines and human peripheral blood mononuclear cells (PBMCs). Overall, we demonstrated that these cellulose-based nanomaterials present very low cytotoxicity in all our tested experimental conditions [20]. Given that these cationic cellulose-based nanomaterials have the potential to be developed as immunomodulators and therefore vaccine nanoadjuvants, it is paramount that all aspects of their interactions with biological systems must be evaluated. The majority of currently utilized vaccines are administered intramuscularly (i.e., direct injection into the skeletal muscle) [15], implying that these nanoadjuvants have the possibility to be in contact with blood and vascular cells. As part of the biomedical application safety assessment of nanoparticles, the blood compatibility assays comprehend a series of tests to verify the interaction between nanomaterials and blood components alongside its consequences including hemolysis [21] and RBC aggregation and morphology [22]. Thus, in this study, we utilized RBC lysis, aggregation, and morphology, as well as cytotoxicity in endothelial cells in dose-response and time course studies to evaluate the blood and vascular compatibility of engineered cationic CNCs. Overall, we demonstrated that unmodified and modified CNCs are compatible with RBC and endothelial cells, and therefore can be further investigated as potential vaccine nanoadjuvants.

## 2. Materials and Methods

### 2.1. Cationic CNCs and Preparation of Their Colloidal Suspensions for Biological Assays

**Scheme 1:**
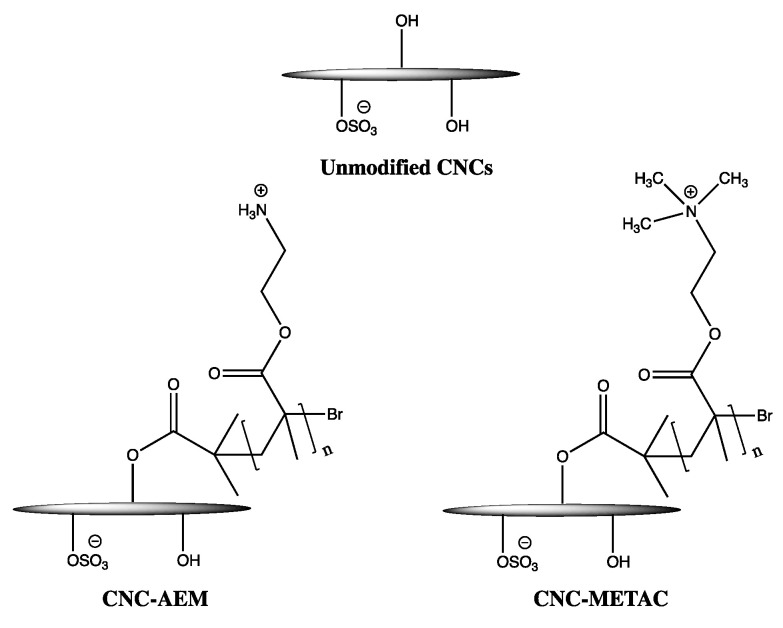
Chemical representation of the cellulose-based nanomaterials used in this study: unmodified CNCs and engineered cationic CNCs (CNC-AEM and CNC-METAC).

The unmodified CNCs used in this study were spray-dried CNCs obtained via sulfuric acid hydrolysis of hardwood pulp that were kindly supplied by InnoTech Alberta Inc. (Edmonton, AB, Canada). The unmodified CNCs possess a negative surface charge due to the presence of the sulfate half-ester groups. CNCs conjugated with the cationic polymers [2-(methacryloyloxy)ethyl]trimethylammonium chloride (METAC) and 2-aminoethyl methacrylate hydrochloride (AEM) were synthesized via surface-initiated single electron transfer living radical polymerization and characterized using analytical, spectroscopy and microscopy techniques as described in our recent publication [20]. The chemical structures of unmodified CNCs and engineered cationic CNCs (also referred to as modified CNCs) are depicted in Scheme 1 and their respective compositions, apparent particle sizes, and zeta potentials are illustrated in Table S1. The cationic CNCs used in this study are CNC-AEM-1A, CNC-AEM-2A, CNC-METAC-1A, CNC-METAC-2A, and CNC-METAC-2B. While CNC-METAC (1A, 1B, and 2A) have the same chemical structures, they differ in terms of their composition based on the amount of initiator and monomer used during the polymerization. For instance, CNC-METAC-1A and CNC-METAC-1B were prepared using the same amount of initiator (5:3 [Br]/[AGU]) but with a different monomer concentration (50:3 and 60:3 [monomer]/[AGU], respectively). CNC-METAC-2B was synthesized with 5:12 [Br]/[AGU]) and 60:3 [monomer]/[AGU] (Table S1). The colloidal suspensions of the unmodified and modified CNCs were prepared at 1 mg/mL in ultrapure water by vortexing for 15 sec followed by sonication (70 output) for 2 min. The sonicated suspensions were filtered using a 0.45 µm polytetrafluoroethylene (PTFE) filter and autoclaved at 121° C 15 psi for 30 min. The sterile CNCs suspensions were aliquoted and kept at −20 °C. For simplicity, the names of the modified cationic CNCs were abbreviated in the figures by omitting “CNC” from their denominations. For full name, chemical structures, and respective physicochemical characteristics, see Table S1 and Scheme 1.

### 2.2. Human Blood Preparation

Human blood containing citrate phosphate double dextrose Solution (CP2D) as an anticoagulant were extracted from Leukotrap blood filters (UVM Health Network-CVPH North Country Regional Blood Center) from healthy blood donors. To retrieve blood cells, the filter was slowly flushed once with a 50 mL syringe filled with air and approximately 15 mL was collected. The blood was diluted 1:10 in sterile calcium and magnesium-free phosphate buffered saline (PBS) to obtain approximately 1.5 mg/mL of total hemoglobin. In a 48-well plate, 225 µL of diluted blood was mixed with 25 µL of CNCs suspensions (final concentrations of 25 and 50 µg/mL), followed by incubation at 37 °C in a 5% CO_2_-supplemented atmosphere for 1, 2, and 24 h before analysis.

#### 2.2.1. Hemolysis Assay

The hemolysis assay was modified using the protocol from the National Cancer Institute (NCI) [23] and as previously published [24]. After the respective period of incubation, the percentage (%) of RBC hemolysis was analyzed by transferring 150 µL of the RBC/CNC mixture into a clean microtube and centrifuging it at 4000 RPM. The supernatant was then reacted with 1 mL of Drabkin solution (Ricca, Arlington, TX, USA) for 10 min RT in the dark, followed by spectrophotometric analysis at 540 nm. To determine the total hemoglobin in each experimental condition, 1% triton was added to 20 μL of the RBC/CNCs mixture for the complete release of hemoglobin prior to the addition to the 1 mL of Drabkin solution and the absorbance was measured at 540 nm using a microplate reader (Synergy H1 Hybrid Multi-Mode, BioTek/Agilent, Winoski, VT, USA). PBS (negative) ultrapure water, Triton 1%, and polyethylene glycol (PEG) 1 mg/mL (positive) were utilized as controls. The absorbance of the Drabkin solution at 540 nm was considered blank and subtracted from the absorbance obtained from all the samples. The percent of RBC lysis was calculated using the following equation.

$$\%\;\mathrm{lysis}=[\frac{\left(\mathrm{Abs}540\;\mathrm{nm}\;\mathrm{of}\;\mathrm{supernatant}s-\mathrm{Abs}540\;\mathrm{nm}\;\mathrm{of}\;\mathrm{blank}\right)}{\left(\mathrm{Abs}540\;\mathrm{nm}\;\mathrm{of}\;\mathrm{suspension}-\;\mathrm{Abs}540\;\mathrm{nm}\;\mathrm{of}\;\mathrm{blank}\right)\;x\;diluition\;factor}\;]\times 100$$

#### 2.2.2. Red Blood Cell (RBC) Morphology and Aggregation

After the treatments, 10 µL of blood/CNCs mix were diluted in 100 µL of sterile calcium and magnesium-free PBS, and were promptly analyzed in a BD Accuri C6 Flow cytometer (BD Biosciences, Franklin Lakes, NJ, USA). At least 20,000 events were collected using a forward scatter channel (FSC) and sideward scatter channels (SCC), and were presented as histograms (FSC vs. count). The data were acquired and analyzed with BD Accuri software. PEG 1 mg/mL (positive control) was utilized for gating the aggregated cells.

For morphological changes and the aggregation of RBC, samples were diluted as described for flow cytometry analysis and observed in an Olympus CKX53 inverted microscope coupled with a DP22 Olympus camera. The Cell Sens (Olympus, Waltham, MA, USA) software was utilized to capture the images in bright field at 400x magnification.

### 2.3. Cell Culture and Experimental Conditions

Human umbilical vein endothelial cells (HUVEC, ATCC Manassas, VA, USA) were cultured in F-12K Medium (Kaighn’s Modification of Ham’s F-12 Medium, ATCC), supplemented with 10% fetal bovine serum (FBS, Gibco/Thermo Fisher Scientific, Waltham, MA, USA), 0.1 mg/mL heparin (MilliporeSigma, Burlington, MA, USA), and 0.3 mg/mL Endothelial Cell Growth Supplement (Corning, Corning, NY, USA). Cells were seeded at 1 × 10^5^ cells/mL in a 96-well plate and cultured at 37 °C in a 5% CO_2_-supplemented atmosphere for at least the overnight before the treatment with 10, 25, 50, and 100 µg/mL of CNCs for 24 or 48 h.

#### Cell Viability Assays

To assess the impact of CNCs on endothelial cell viability, the MTT assay (3-(4,5-dimethylthiazol-2-yl)-2,5-diphenyltetrazolium bromide (Sigma)) and Neutral Red assay (NR, Sigma) were utilized. These assays have different approaches to assess cell viability but both focus on organelle function. The MTT assay assesses the conversion of the water-soluble MTT (yellow) into a water-insoluble formazan (purple/blue), mainly by mitochondrial dehydrogenases [24]. The NR assay is based on the ability of viable cells to incorporate and bind the neutral red dye in the lysosomes [25]. In both assays, the intensity of the color is directly proportional to cell viability. After treatments, the medium from the HUVEC culture was removed and 100 µL of fresh culture medium containing 500 µg/mL of MTT or 50 µg/mL of NR was added to each well. The cells with the MTT or NR loading medium were incubated at 37 °C in a 5% CO_2_-supplemented atmosphere. After 30 min, the respective loading medium was removed and the attached cells were gently washed once with PBS. To solubilize the formazan crystals, 100 μL/well of dimethyl sulfoxide (DMSO) was added and the absorbance was measured at 570 nm using a microplate reader (Synergy H1 Hybrid Multi-Mode, BioTek/Agilent). To extract NR dye from the lysosomes, 100 μL of acidified ethanol (1% glacial acetic acid, 50% ethanol) was added and the plate was placed on the plate shaker for ~10–15 min with protection from light. The absorbance at 540 nm and 690 nm was measured in the microplate reader within 60 min from adding the NR Desorb solution. For calculations, absorbance at 690 nm was subtracted from 540 nm. The non-treated cells (control) were considered 100% viable cells in both the MTT and NR assays. For statistical significance, both cell viability assays were repeated at least 3 times in triplicates.

### 2.4. Statistical Analysis

The data were statistically analyzed by using the two-way analysis of variance (ANOVA) test followed by a comparison test using GraphPad Prism 8.2 software. Multiple comparison tests and statistical significance are indicated in the legend of the respective figures.

## 3. Results and Discussion

### Blood Compatibility Assay

**Figure 1:**
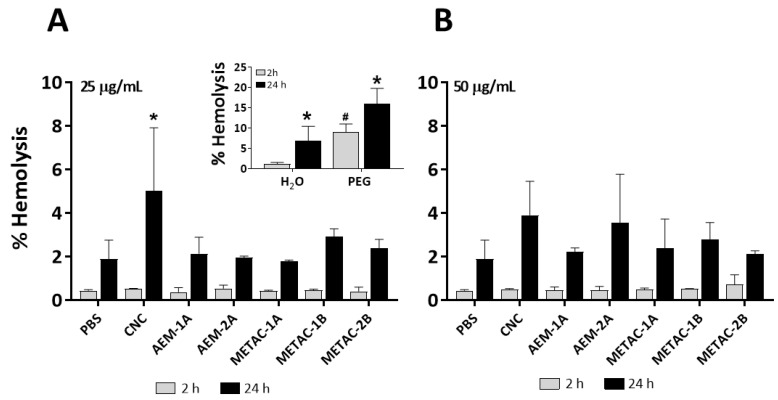
Hemolytic activity of unmodified and modified CNCs in human red blood cells (RBC). Percentage of hemolysis induced by 25 µg/mL (**A**) and 50 µg/mL (**B**) after 2 h (gray bars) and 24 h (black bars) of exposure in diluted human blood with respective concentrations of CNCs. PEG 1 mg/mL, ultra-pure H_2_O, and Triton-X-100 1% (100% hemolysis, not shown) were used as positive controls (insert in **A**) and PBS as negative controls (**A**,**B**). * *p* < 0.05 vs. PBS 24 h; **#** *p* < 0.05 vs. PBS 2 h (Tukey’s).

First, we evaluated the interaction of unmodified CNCs and the respective cationic derivatives with human RBC, as these nanomaterials will be in contact with RBC when entering the circulatory system. Red blood cells (RBC) constitute almost half of blood volume [25] and therefore are important cells to determine the hemocompatibility of a nanomaterial. Initially, we assessed the capability of unmodified and modified CNCs to cause RBC lysis. Hemolysis refers to the damage of red blood cells leading to the release of intracellular content. A percent hemolysis less than 2 means that the nanoparticle is not hemolytic; 2–5% hemolysis means that the nanoparticle is slightly hemolytic; and >5% hemolysis means that the test sample is hemolytic [23]. Most of the CNCs demonstrated none or just slight hemolytic activity according to the definition above. In addition, we did not observe differences in hemolytic activity between the PBS (negative control) and any of the two concentrations of CNCs at a short period of exposure, namely 1 h (Figure S1) and 2 h (Figure 1A,B). As expected, triton 1% showed 100% hemolysis (data not shown) and PEG 1 mg/mL induced significant hemolysis with 2 and 24 h treatment, whereas ultrapure water only showed hemolytic activity at 24 h of incubation (Figure 1A, insert). At 24 h of exposure, however, 25 µg/mL of unmodified CNCs induced hemolysis at the threshold (5% or greater), notably significantly greater than the PBS (Figure 1A, black bars). The hemolytic effect of the unmodified CNCs on the RBC lysis could be attributed, at least in part, to the surface chemistry and reactivity between unmodified and modified CNCs. Unmodified CNCs displayed a negative surface charge (−34.8 ± 2.16 mV) and the derivatized CNCs showed a cationic surface charge ranging between +31.8 ± 2.89 and +45.0 ± 1.44 mV [20]. In addition to the charge differences, the reactivity of unmodified CNCs is also different from the derivatized counterparts. The unmodified CNCs contained greater amounts of hydroxyl and sulfate half-ester functional groups, and this abundance could lead to greater interactions with RBC membranes. For instance, hydroxyl moieties on silica NPs have been implicated in the hemolytic activity of silica as this functional group promotes interactions with cell membranes [26]. Additionally, the significant hemolytic effect of the unmodified CNCs occurred at a lower concentration, rather than higher. This apparent unexpected result could be due to the capability of unmodified CNCs to agglomerate at higher concentrations and therefore impact their interaction with cellular membranes. The degree of agglomeration of CNCs in cell culture media is among the several factors that can impact the results of biocompatibility assays [27].

**Figure 2:**
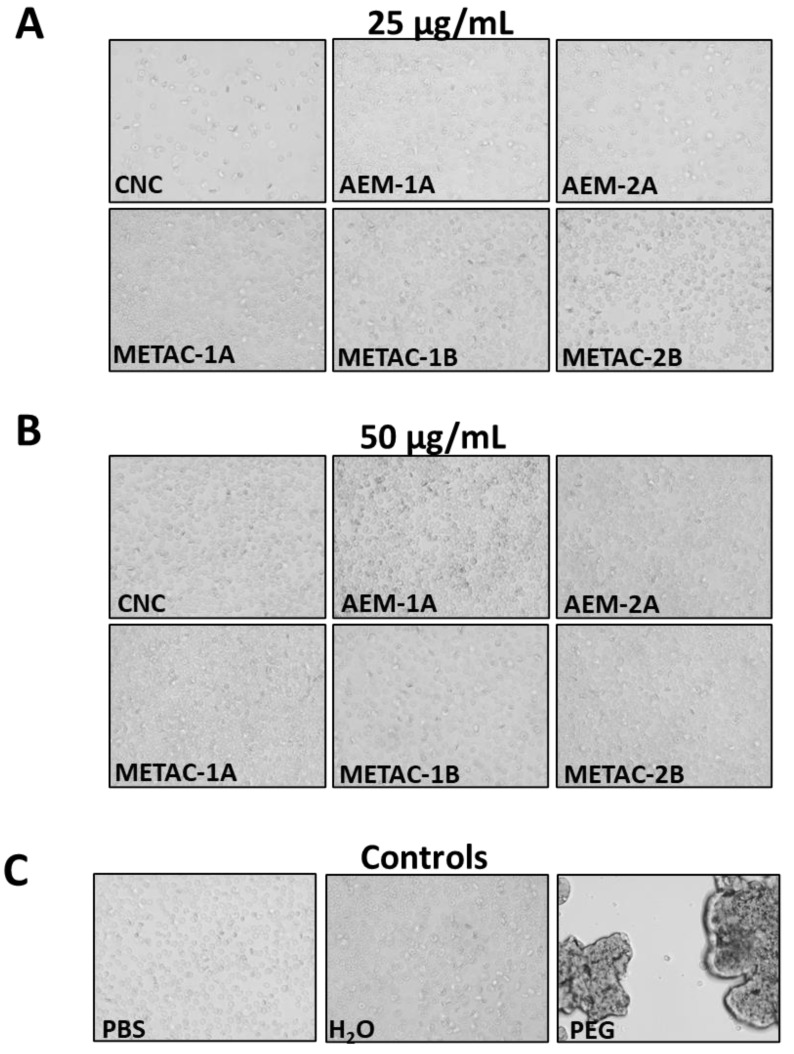
Effect of unmodified and modified CNCs on RBC morphology and aggregation. Diluted human blood was treated for 2 h with (**A**) 25 µg/mL or (**B**) 50 µg/mL of unmodified and modified CNCs, or with controls, (**C**) PBS, ultrapure H_2_O, and PEG 1 mg/mL. The pictures were captured using a bright-field inverted microscope (400×).

**Figure 3:**
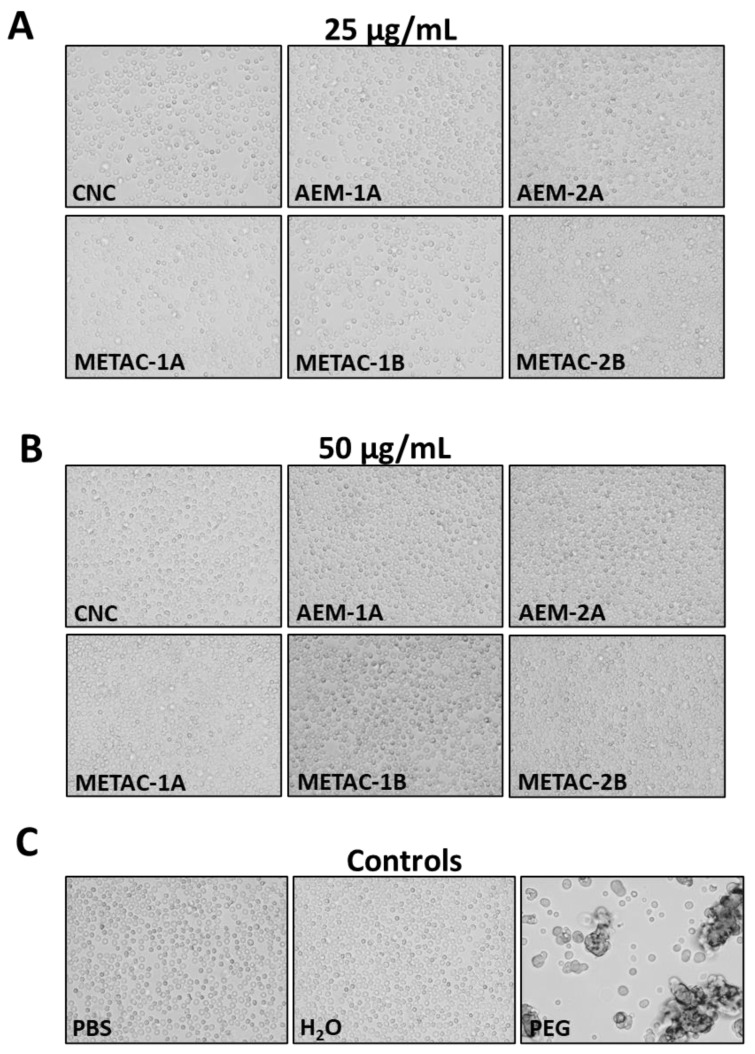
Effect of unmodified and modified CNCs on RBC morphology and aggregation. Diluted human blood was treated for 24 h with (**A**) 25 µg/mL or (**B**) 50 µg/mL of unmodified and modified CNCs, or with controls, (**C**) PBS, ultrapure H_2_O, and PEG 1 mg/mL. The pictures were captured using a bright-field inverted microscope (400×).

**Figure 4:**
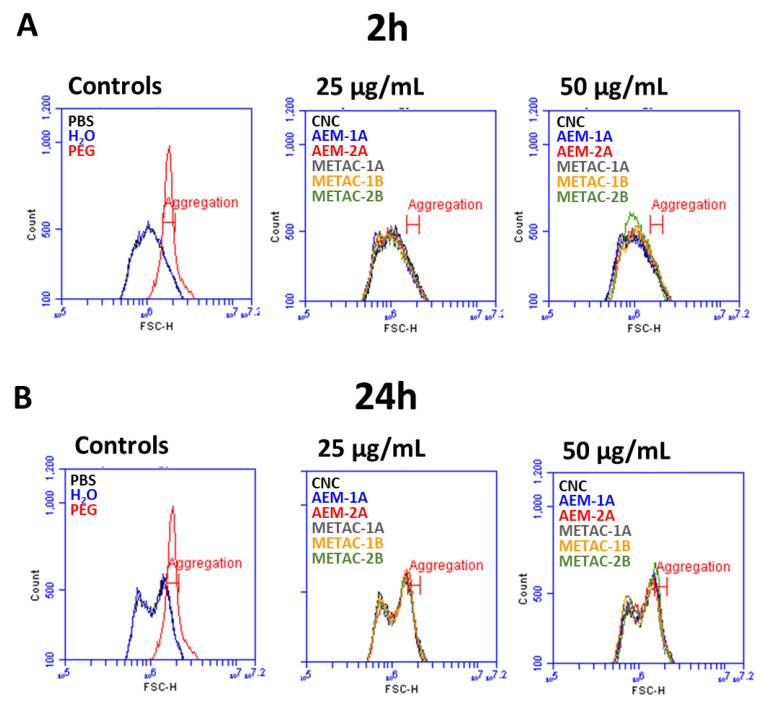
Effect of unmodified and modified CNCs on red blood cell (RBC) aggregation. Diluted human blood were treated for 2 h (**A**) or 24 h (**B**) with controls, PBS, ultrapure H_2_O, and PEG 1 mg/mL (2 h only), or with 25 µg/mL or 50 µg/mL of unmodified and modified CNCs. After respective treatments, RBC aggregation was accessed by flow cytometry. The histograms displayed the number of events vs. the FSC-H (forward light scatter) high channel (size estimation). The aggregation of RBC was gated using PEG 1 mg/mL (red, 2 h) for both 2 and 24 h time points (**A**,**B**).

In parallel, we also investigated the possibility of unmodified CNCs and their cationic derivatives to cause RBC aggregation or changes in the RBC morphology using cell imaging and flow cytometry approaches. RBC aggregation and morphology together with RBC lysis are important parameters to assess the biocompatibility of nanomaterials [22]. None of the CNCs induced RBC aggregation at short (Figure 2A,B) or long exposure (Figure 3A,B), despite their cationic surface charges [20] and cationic polymers such as polyethyleneimine (PEI) that are well known to cause RBC aggregation [28]. The positive control, PEG 1 mg/mL, induced strong RBC aggregation in both short and long exposure (Figure 2 and Figure 3C, respectively). The flow cytometry data confirmed what was observed in the cell imaging analysis. Typically, side scatter (SSC) signals are attributed to cellular internal structure and organelles, representing cellular granularity, and the forward scatter (FSC) signal is proportional to the diameter of the cell, representing the size of the cells [29]. None of the cationic CNCs or unmodified CNCs caused RBC aggregation at short or longer exposure (Figure 4A,B, respectively). PEG 1 mg/mL as a positive control was used to gate the “aggregation” behavior of the RBC. Although there was no major difference between RBC exposed to PBS (negative control) and cells exposed to unmodified and modified CNCs, we observed that the histogram profile had changed over time. At longer exposure (Figure 4B), the histogram displays two peaks for all the conditions, instead of one peak with 2 h of exposure (Figure 4A). RBC aggregation is expected to increase the FSC by shifting the histogram to the right, as observed in the positive control PEG (Figure 2 and Figure 3C). The histogram of RBC exposed to 50 µg/mL of CNC-METAC-2B showed a mild increase in the number of events (counts) in the same region but not a right shift as expected for the increasing size of the cells which is an indication for RBC aggregation. This effect is more evident at 2 h (Figure 4A, right panel, green line) than at 24 h (Figure 4B, right panel, green line) of treatment. We did not observe differences in cellular granularity in all the conditions tested (Figure S2).

**Figure 5:**
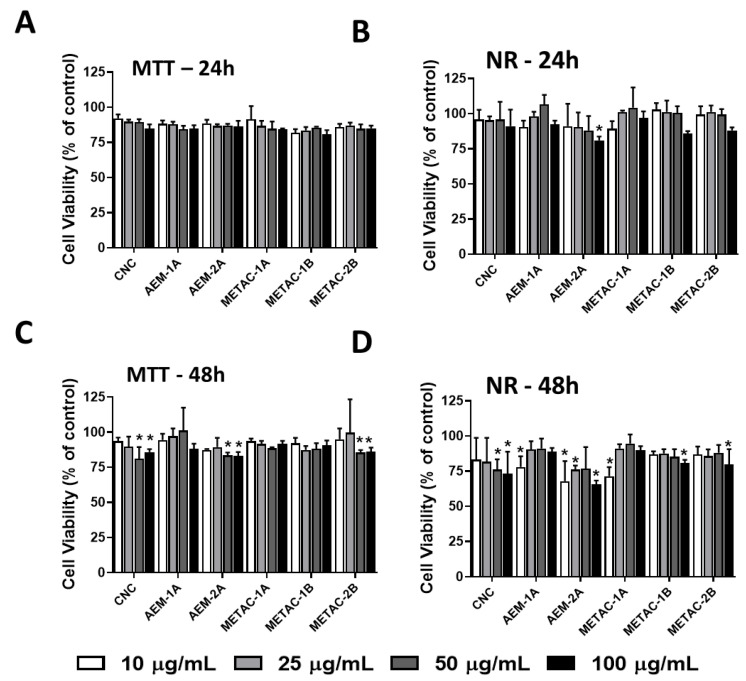
Effect of unmodified and modified CNCs on the cell viability of the HUVEC culture assessed by MTT (**A**,**C**) and NR (**B**,**D**) assays. After 24 h (**A**,**B**) or 48 h (**C**,**D**) of treatment, cell viability was determined spectrophotometrically using the respective assays. Representative of three independent experiments in triplicates. * *p* < 0.01 compared to the control, non-treated cells, (100% cell viability, Dunnett’s).

The surface chemistry and the morphology (size, shape, and state of aggregation) of nanoparticles that are in contact with the cells are important physicochemical aspects that drive the cytotoxicity of these nanomaterials [26]. Considering the growing interest in nanomaterials for biomedical applications, assessment of the toxicity in cell-based assays has become a crucial part of the characterization of nanomaterials [30]. In addition to the analysis of the impact of these cellulose-based nanomaterials on RBC and as part of the assessment of the biocompatibility of CNCs for potential biomedical applications, we chose to evaluate the effect of unmodified and engineered cationic CNCs on the cell viability of endothelial cells. Endothelial cells form a single cell layer that lines all blood vessels and regulates exchanges between the bloodstream and the surrounding tissues [31]. They are relevant in this context as these nanomaterials can reach systemic circulation and potentially be in contact with these cells; thus, they have been used to assess the biocompatibility of nanomaterials [28]. To evaluate the cytotoxicity of the cationic CNCs, we chose to perform MTT and NR assays because these low cost and reproducible assays are largely used for the screening of the cytotoxicity of compounds in general with potential biomedical applications. Most of the CNCs materials tested did not show a statistically significant decrease in the viability of HUVECs with 24 h of treatment, as demonstrated by MTT (Figure 5A) or NR (Figure 5B) assays. The exception is CNC-AEM-2A that at the highest concentration showed approximately a 20% decrease of cell viability using the NR assay (Figure 5B). The overall result is consistent with our previous work, in which we demonstrated that the same nanomaterials did not cause a decrease in the viability of human peripheral blood mononuclear cells (PBMCs) [20]. At 48 h of treatment, all nanomaterials, including unmodified CNCs, showed some degree of decrease in cell viability in both assays (Figure 5C,D). However, we did not observe a relevant dose–response relationship, which is the central concept in toxicology [32]. In general, this data is also consistent with the hemolytic effect of these compounds. The lack of a relevant dose-response decrease in cell viability is not a surprise as they also did not show relevant hemolytic activity. It has been suggested that hemolytic activity is generally associated with the cytotoxicity of nanoparticles [26].

## 4. Conclusions

In conclusion, we have assessed the vascular and blood compatibility of a series of engineered cationic CNCs. The interaction of both unmodified CNCs and cationic CNCs with human RBC concerning to RBC aggregation or changes in the RBC morphology was evaluated using cell imaging and flow cytometry approaches. We also assessed the effect of unmodified and engineered cationic modified CNCs on the cell viability of endothelial cells using MTT and NR assays. Overall, our results indicated that unmodified and engineered cationic CNCs are compatible with RBC as well as with endothelial cells. The findings of this study, together with the immunomodulatory properties of these cationic CNCs previously published, support the development of engineered biocompatible cationic CNCs for biomedical applications, in particular as vaccine nanoadjuvants.

## Data Availability

The data presented in this study are available in the main manuscript and in the Supplementary Materials.

## Supplementary Materials

The following are available online at https://www.mdpi.com/article/10.3390/nano11082072/s1: Table S1: Composition of cationic CNCs as well as their respective zeta potential and apparent particle sizes; Figure S1: Hemolytic activity of unmodified and modified CNCs at 1 h of exposure; and Figure S2: Side scatter flow cytometer histograms of blood exposed for 2 and 24 h at different concentrations of unmodified and modified CNCs.
