# Supplementary material for: Vascular and Blood Compatibility of Engineered Cationic Cellulose Nanocrystals in Cell-Based Assays
Source: Nanomaterials (Basel). 2021 Aug 15;11(8):2072. doi: 10.3390/nano11082072 (PMC8399684; doi:10.3390/nano11082072)
Supplement: Supplementary file 1 [file nanomaterials-11-02072-s001.zip › nanomaterials-1316998-supplementary.pdf]

Supporting Information

Vascular and blood compatibility of engineered cationic cellulose nanocrystals in cell-based assays

Alexandre Bernier[1], Tanner Tobias[1], Hoang Nguyen[1], Shreshth Kumar[1], Beza Tuga[1], Yusha Imtiaz[1], Christopher W. Smith[1], Rajesh Sunasee [1,*] and Karina Ckless [1,*]

[1]Department of Chemistry, State University of New York at Plattsburgh, Plattsburgh, New York, USA, 12901.

*Correspondence: kckle001@plattsburgh.edu (K.C.) rajesh.sunasee@plattsburgh.edu (R.S.)

Table S1: Cationic CNCs used in this study prepared with different compositions by varying the molar ratios of [Br]/[AGU] and [monomer]/[AGU] and their apparent particle sizes and zeta potentials[1]

Cationic CNCs	[Br]/[AGU]	[Monomer/ [AGU]	Apparent Particle Size (nm)	Zeta Potential (mV)
CNC-METAC-1A	5:3	50:3	123.4 ± 1.32	+31.8 ± 2.89
CNC-METAC-1B	5:3	60:3	136.2 ± 1.50	+44.9 ± 3.93
CNC-METAC-2B	5:12	60:3	203.2 ± 2.66	+38.2 ± 0.94
CNC-AEM-1A	5:3	50:3	172.0 ± 9.08	+45.0 ± 1.44
CNC-AEM-2A	5:12	50:3	215.3 ± 2.86	+41.4 ± 3.15g

Note: For the actual Figures in the manuscript, the names of the cationic CNC samples were shortened by omitting the word "CNC-" for labeling simplicity (for example: CNC-AEM-1A becomes AEM-1A, CNC-METAC-1A becomes METAC-1A etc.)

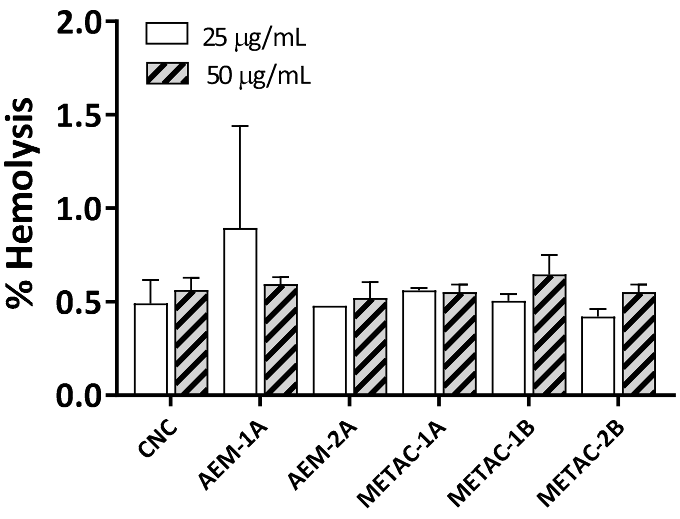

Figure S1: Hemolytic activity of unmodified and modified CNCs in human blood. Percentage of hemolysis induced by 25 μg/mL or 50 μg/mL after 1h of exposure of diluted whole human blood with respective concentrations of CNCs. For simplicity, the name of the modified CNCs were abbreviated by omitting "CNC" from their denominations. For full name and respective physicochemical characteristics see Table S1 and Scheme 1.

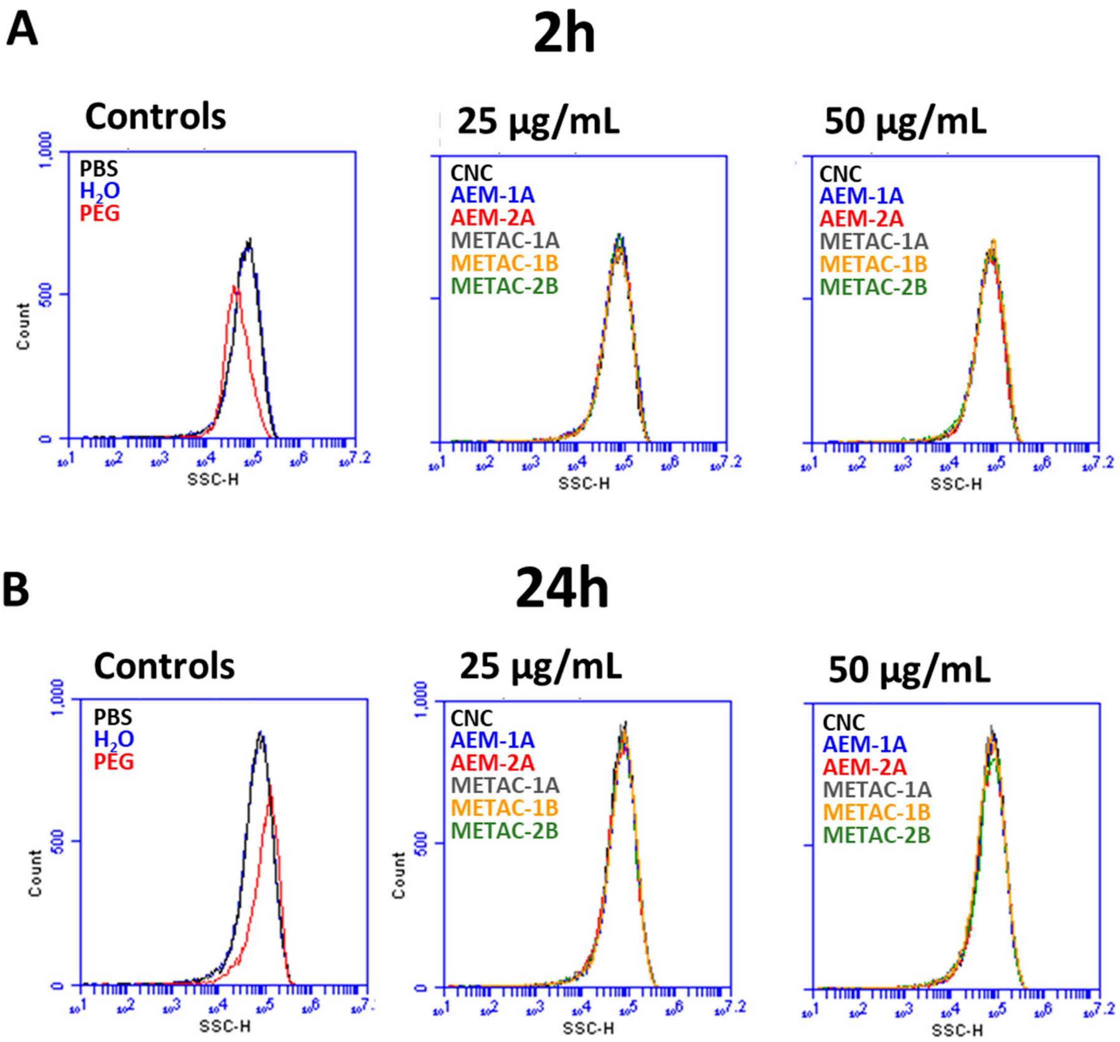

Figure S2: Effect of unmodified and modified CNCs on cellular granularity. Diluted whole human blood were treated for 2h (A) or 24h (B) with controls, PBS, ultrapure H_2O and PEG 1 mg/mL (2h only) or 25 µg/mL or 50 µg/mL of unmodified and modified CNCs. After respective treatments the samples were submitted to flow cytometry analysis and the plots relating number of events and side light scatter high channel (SSC-H) distribution (granularity) are displayed. For simplicity, the name of the modified CNCs were abbreviated by omitting "CNC" from their denominations. For full name and respective physicochemical characteristics see Table S1 and Scheme 1.

Reference

1. Imtiaz Y, Tuga B, Smith CW, Rabideau A, Nguyen L, Liu Y, Hrapovic S, Ckless K, Sunasee R. Synthesis and Cytotoxicity Studies of Wood-Based Cationic Cellulose Nanocrystals as Potential Immunomodulators. *Nanomaterials*, **2020**, 10(8):1603. doi: 10.3390/nano10081603.
